# Single-step fabrication of fibrous Si/Sn composite nanowire anodes by high-pressure He plasma sputtering for high-capacity Li-ion batteries

**DOI:** 10.1038/s41598-023-41452-3

**Published:** 2023-09-08

**Authors:** Giichiro Uchida, Kodai Masumoto, Mikito Sakakibara, Yumiko Ikebe, Shinjiro Ono, Kazunori Koga, Takahiro Kozawa

**Affiliations:** 1 Faculty of Science and Technology, Meijo University, 1-501 Shiogamaguchi, Tempaku-Ku, Nagoya, 468-8502 Japan; 2 Graduate School and Faculty of Information Science and Electrical Engineering, Kyushu University, 744 Motooka, Nishi-Ku, Fukuoka, 819-0395 Japan; 3 Joining and Welding Research Institute, Osaka University, 11-1 Mihogaoka, Ibaraki, 567-0047 Japan

**Keywords:** Nanowires, Batteries, Synthesis and processing

## Abstract

To realize high-capacity Si anodes for next-generation Li-ion batteries, Si/Sn nanowires were fabricated in a single-step procedure using He plasma sputtering at a high pressure of 100–500 mTorr without substrate heating. The Si/Sn nanowires consisted of an amorphous Si core and a crystalline Sn shell. Si/Sn composite nanowire films formed a spider-web-like network structure, a rod-like structure, or an aggregated structure of nanowires and nanoparticles depending on the conditions used in the plasma process. Anodes prepared with Si/Sn nanowire films with the spider-web-like network structure and the aggregated structure of nanowires and nanoparticles showed a high Li-storage capacity of 1219 and 977 mAh/g, respectively, for the initial 54 cycles at a C-rate of 0.01, and a capacity of 644 and 580 mAh/g, respectively, after 135 cycles at a C-rate of 0.1. The developed plasma sputtering process enabled us to form a binder-free high-capacity Si/Sn-nanowire anode via a simple single-step procedure.

## Introduction

Electric vehicles powered by Li-ion batteries are increasingly replacing gasoline-powered vehicles toward the realization of a carbon–neutral society, and the development of high-performance Li-ion batteries that have high capacity for long-distance driving, high power for rapid charging, and low degradation for long-term use is encouraged worldwide^1–3^. Group-IV Si^4–10^, Ge^11–20^, and Sn^21,22^ are promising anode materials for Li-ion batteries because they have very high theoretical capacities of 4200, 1600, and 993 mAh/g, respectively, which are 2.6–11 times higher than the 372 mAh/g capacity of commercial carbon. However, the high-capacity Si, Ge, and Sn materials suffer large volume expansion during Li alloying reactions, and repeated cycling leads to material pulverization from the current collector, resulting in performance degradation.

Researchers have used various approaches to improve the cycling stability of the highest-capacity Si anodes^23–28^. One of the most promising approaches is to create different morphologies such as porous structures, nanoparticles, and nanowires, which exhibit better physical and mechanical stability against volume expansions. In general, nanomaterials uniformly expand during Li alloying because of their large surface-to-volume ratio, reducing physical stress and suppressing material pulverization. In particular, Si anodes with a one-dimensional (1D) nanowire morphology have shown enhanced electrochemical properties because their 1D structure better accommodates the colossal volume changes without physically fracturing, where the physical strain in the nanowires is facilely relaxed by radial and axial expansion^25–28^.

Si nanowires are prepared through one of two basic approaches: a bottom-up or top-down process^9^, as summarized in review papers^29,30^. In the case of bottom-up processes, Si nanowires have been fabricated on substrates via (1) physical vapor deposition (PVD) from a solid Si source using laser ablation^31–34^, vacuum ohmic-heating^35^, or thermal plasma^36^, (2) chemical vapor deposition (CVD) from SiH_4_ gas using vacuum ohmic-heating^37^ or low-temperature plasma^38^, and (3) a solution-based synthesis^39^. In all of these bottom-up processes, vapor–liquid–solid (VLS) growth is a common mechanism for the synthesis of Si nanowires. Metallic nanodots such as Au are first formed on the substrate, and Si atoms are subsequently supplied to the nanodots, which function as a molten metal catalyst^40^. When Si is supersaturated in the metal alloy, it grows as a nanowire at the Si/metal catalyst interface.

Our focus is on the development of a simplified Si VLS growth process using a plasma technique, which can potentially enable the fabrication of films at high speed over large areas, as required for industrial applications. Nguyen et al. reported Au-seeded Si nanowire film growth by plasma CVD, where a Au catalyst was first deposited onto a stainless steel substrate, followed by SiH_4_/H_2_ plasma deposition for the VLS-growth of Si nanowires at a substrate temperature of 420 °C^38^. A Li-ion battery with a Si-nanowire anode maintained a high capacity of 3100 mAh/g for the first 40 cycles.

Recently, Tanaka et al. reported the single-step production of Si nanorods from a powder feedstock using thermal-plasma PVD, where Si and Cu powders were introduced into a thermal plasma and a large number of VLS-grown Si nanorods were recovered as product particles^36^. Tanaka et al. coated a slurry containing the synthesized Si nanorods onto metal current collectors and sintered them to form a rigid anode film. A Li-ion battery with a Si-nanorods anode exhibited a capacity of 500 mAh/g and good cycling capability for 100 charge–discharge cycles.

In the present study, we propose an alternative plasma bottom-up process to fabricate Si-nanowire films for Li-ion-battery anodes using plasma sputtering PVD. The important advantage of our plasma sputtering PVD is that it employs a simple single-step procedure that enables the direct fabrication of Si-nanowire films on a metal current collector without metal catalyst nanodots or heating of the substrate. Our plasma PVD process, which does not use an organic solvent, differs substantially from other methods: (1) Our method enables direct anode film formation on a current collector without a binder, whereas other methods use a slurry coating process that includes nanowires; (2) unlike other PVD processes such as laser ablation, our method enables film formation at scales as large as meter-scale; and (3) our method uses a simple control process for a 1D nanowire structure in single-step procedure without precise temperature control, whereas other CVD processes use SiH_4_ gas and a Si solution. In addition, to enhance the electrochemical reaction related to Li^+^ ions in a high-capacity Si anode, another group IV element, Sn, was added to the Si anode film in the present study^41^. Metallic Sn has a high theoretical Li storage capacity of 993 mAh/g, and its electrical conductivity (10^4^ S/cm) is greater than that of the intrinsic semiconductor Si (10^−6^ S/cm). The high performance of Sn as an anode material has been summarized in review papers^21,42^. The plasma sputtering process developed in the present study enables the continuous formation of a binder-free Si/Sn nanowire film, making it potentially applicable for the roll-to-roll fabrication of 1D nanowire electrodes.

## Results and discussion

### Effects of the sputtering-target material and discharge gas on the Si nanostructure

**Figure 1:**
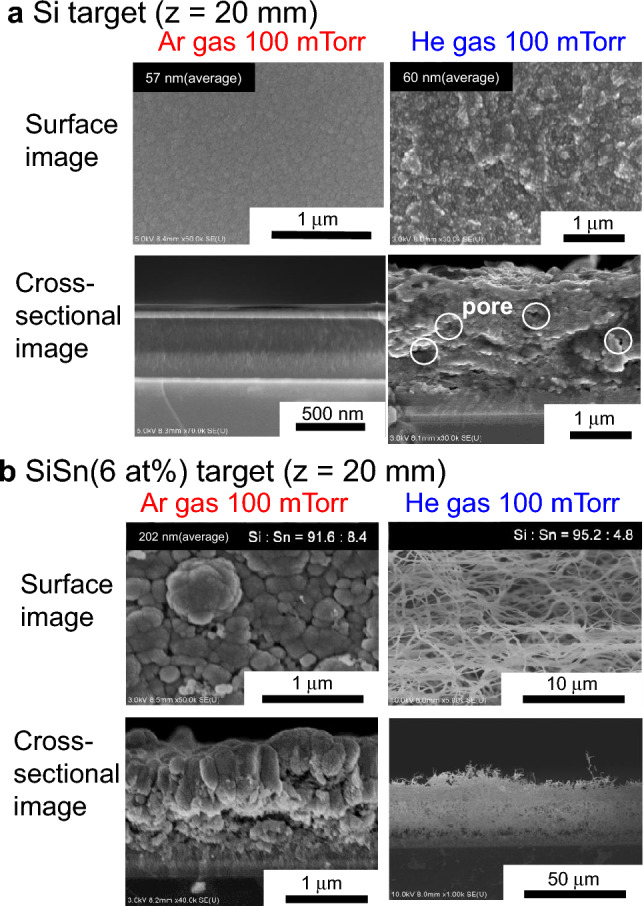
Effects of sputtering-target material and discharge gas on Si nanostructured anodes. SEM surface and cross-sectional images of films deposited at a He or Ar gas pressure of 100 mTorr, where the distance between the sputtering target and substrate was 20 mm: (**a**) sputtering target of Si. (**b**) Sputtering target of SiSn with a Sn content of 6 at%.

Figure 1a, b show surface and cross-sectional scanning electron microscopy (SEM) images of films deposited via 100 mTorr high-pressure Ar and He plasma sputtering with an intrinsic Si target and a SiSn target with a Sn content of 6 at%, respectively. The film morphology markedly differed depending on the discharge gas. Nanostructured grains are observed on the surface of the Si films deposited with Ar discharge gas (left image of Fig. 1a), and a dense film structure is observed in the cross-sectional SEM image. However, the Si nanoparticle film deposited using He as the discharge gas exhibited a much greater roughness; the average nanoparticle size was estimated to be 60 nm from more than 20 randomly selected particles in the SEM image. A porous structure with abundant pores was clearly observed in the cross-sectional SEM image of the nanoparticle film (right image of Fig. 1a). We evaluated the porosity of the deposited Si nanoparticle films. The mass density *ρ* of the deposited films was measured and subsequently compared with the bulk Si density of 2.32 g/cm^3^. The porosity, calculated as ((2.32 − *ρ*)/2.32)×100 (%), was as high as 17.6%. Here, a *ρ *of 1.91 g/cm^3^ was estimated from the mass and volume of the deposited Si nanoparticle film; the film mass was derived from the difference in the substrate mass before and after the deposition process, and the film volume was estimated from the product of the deposition area and the film thickness obtained from the cross-section SEM image.

**Figure 2:**
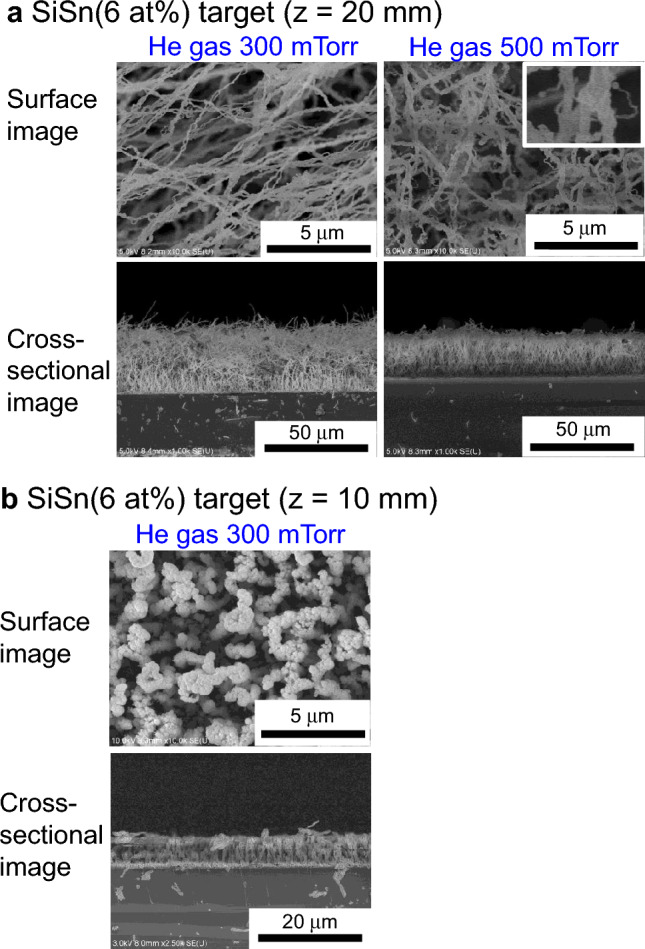
Effect of He gas pressure and sputtering-target/substrate distance on Si nanowire anodes. SEM surface and cross-sectional images of films deposited using a SiSn sputtering target with a Sn content of 6 at%. (**a**) He gas pressure of 300 and 500 mTorr; the distance between the sputtering target and substrate was 20 mm. (**b**) He gas pressure of 300 mTorr; the distance between the sputtering target and substrate was 10 mm.

For the SiSn films in Fig. 1b, we observed a distinct transition from nanoparticle films to 1D nanowire films as a result of changing the discharge gas. Porous nanoparticle films were fabricated under Ar discharge gas, where the average nanoparticle size was as large as 202 nm and a cauliflower-like shape due to particle aggregation was observed. By comparison, in the film prepared under He discharge gas (right image of Fig. 1b), abundant Si nanowires longer than 10 μm were deposited in a wiggly and tangled form on the substrate in a single-step procedure, where the average diameter was 287 nm and the Sn content in the Si-nanowire films was 4.8%, as determined by SEM–energy-dispersive X-ray spectroscopy (EDX) analysis. As the He-gas pressure was increased from 100 to 300 and 500 mTorr, the diameter of the Si nanowires decreased from 287 to 155 and 136 nm, respectively (Fig. 2a). The thin Si nanowires exhibited a more fibrous form with a spider-web-like network. In the fibrous film deposited at 500 mTorr, numerous thinner wires were observed to branch from a main nanowire and a small sphere was observed at the top of the thinner wire (see the magnified region of the 500 mTorr SEM image in Fig. 2a). Interestingly, we also observed that Si nanowires vertically align on the substrate, as evident in the 300-mTorr cross-sectional image (Fig. 2a).

We also reduced the target–substrate distance *z* from 20 to 10 mm. When the *z* distance was reduced to 10 mm, the wire morphology changed from the fibrous form to the rod-like form with a larger average diameter of 605 nm and a longer inter-rod distance of a few micrometers (Fig. 2b). The SEM image of the Si rod-like film surface shows a cauliflower-like pattern and indicates that the rods are composed of aggregated nanoparticles. On the basis of morphological analysis by SEM, we identified three nanostructure patterns: (1) a porous nanoparticle film, (2) a fibrous 1D nanowire film with the spider-web-like network, and (3) a 1D rod-like film composed of nanoparticles. Thus, in the present study, we successfully fabricated 1D nanostructured films in a single step under the special plasma sputtering conditions of a SiSn(6 at%) target and high-pressure He discharge gas.

**Figure 3:**
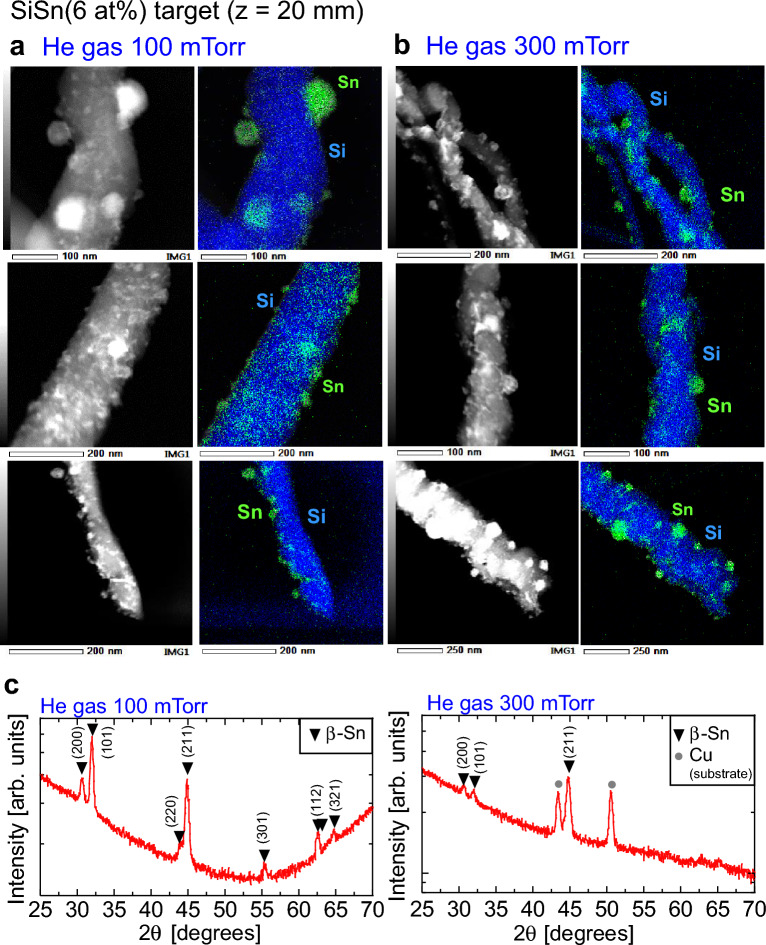
Structure analysis of Si/Sn composite nanowires by TEM and EDX. Nanowires were fabricated using a SiSn sputtering target with a Sn content of 6 at% and a sputtering-target-to-substrate distance of 20 mm. (**a**) TEM images of nanowires and TEM–EDX mapping images of Si (blue) and Sn (green); the He gas pressure was 100 mTorr. (**b**) TEM images of nanowires and TEM–EDX mapping images of Si (blue) and Sn (green); the He gas pressure was 300 mTorr. (**c**) XRD patterns of Si/Sn nanowires fabricated at a He gas pressure of 100 and 300 mTorr.

To clarify the detailed structure of the Si nanowires, we conducted TEM analysis. Figure 3a, b show TEM images of nanowires fabricated under He discharge-gas pressures of 100 and 300 mTorr, respectively. Elemental mapping images of wires were acquired by EDX, where blue and green indicate Si and Sn atoms, respectively. As evident in the TEM image of Fig. 3a (top), nanoparticles with sizes of 40–50 nm are arranged on the wire surface; the nanoparticles were shown by EDX analysis to be composed of Sn (green). Many Sn nanoparticles smaller than 40–50 nm were also observed on the Si core surface (blue), and the nanowire exhibited a core–shell structure consisting of a Si core covered with a Sn shell layer (middle image of Fig. 3a). We also carefully observed the top of the nanowire and found that the nanospheres were not concentrated at the tip but rather distributed randomly on the Si core surface (bottom image of Fig. 3a).

For the higher-pressure condition of 300 mTorr, thinner nanowires were found tangled with each other (top image of Fig. 3b). Interestingly, we observed that the thin nanowires had a neck structure composed of Si and Sn spherical parts and that the nanoparticles appeared to be arranged in a row, resembling beads (middle image of Fig. 3b). Similar to the nanowires formed under the 100 mTorr condition, nanowires formed under the 300 mTorr condition did not have nanospheres concentrated at their tip (bottom image of Fig. 3b).

Details of the crystal structure of the Si/Sn composite nanowire were obtained by X-ray diffraction (XRD) analysis. Numerous sharp signals were observed in the 2*θ* range from 25° to 70° in the patterns of both the 100- and 300-mTorr films (Fig. 3c); these peaks were assigned to the crystalline β-Sn phase, which exhibits high electrical conductivity. However, XRD peaks attributable to crystalline Si were not detected and the Si core was identified as amorphous. It is reasonable that the crystallization of Si under our experiment conditions without a heated substrate is difficult because of the melting point of Si (1410 °C), which is much higher than that of Sn (231 °C). On the basis of the whole structure analysis, we concluded that the nanowires consist of an amorphous Si core with a high Li-ion capacity and a crystalline Sn shell with high electrical conductivity.

### Growth mechanism of Si nanowires

**Figure 4:**
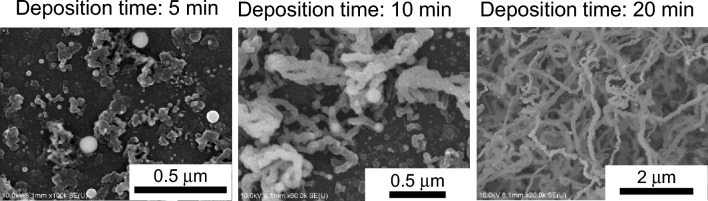
Effect of deposition time on Si/Sn composite nanowire anodes. SEM surface images of films deposited for 5, 10, and 20 min. The distance between the sputtering target and substrate was 20 mm under the condition of a He gas pressure of 300 mTorr; the Sn content of the SiSn sputtering target was 6 at%.

To understand the growth mechanism in the single-step nanowire fabrication process that does not involve complicated preparation of metal catalyst nanodots, we conducted two additional experiments on the films to evaluate (1) the effect of the deposition time and (2) the effect of the Sn content. First, Fig. 4 shows SEM surface images of films deposited for 5, 10, and 20 min. In the SEM image of the film deposited for 5 min, spherical particles with various sizes, which appear white in the image, were observed. At 10 min after the start of deposition, wiggly shaped wires grew sparsely on the substrate and the spherical particles appeared to be the starting point of nanowire growth via a common VLS mechanism. After 20 min of deposition, a fibrous nanowire film as thick as ~ 20 μm was observed. During initial particle formation on the film, the solubility of Sn in the Si material is low according to the binary phase diagram; Sn therefore precipitates as dots from the SiSn mixed film^43–46^. The particles could be in the droplet state in our high-pressure He sputtering system because the thermal conductivity of He gas is one order of magnitude higher than that of Ar gas. Under He, the heat of the sputtering target is therefore efficiently transferred to the reaction field both in the gas plasma and on the substrate surface. To confirm this effect, we roughly measured the substrate surface temperature with a thermocouple probe, where the plasma and the He neutral gas directly flowed to the small tip surface of the sensor placed on the substrate. The thermocouple temperatures rapidly increased with increasing deposition time until the deposition time reached 4 min (i.e., 254 °C under He discharge and 98 °C under Ar discharge gas) and then remained almost constant. At the shorter *z* distance of 20 mm under highly thermally conductive He gas, the temperature of the sensor tip increased, which we attributed to (1) efficient heat transfer from the sputtering target at an RF input power of 15.7 W/cm^2^ (80 W) via the flowing He gas and (2) physical and chemical reactions involving impinging ions from the plasma on the surface^47^. The melting point of Sn is as low as 231 °C, and the increase in temperature to greater than 254 °C explains the Sn droplet formation from a thermal perspective. In addition, the melting point of the nanoparticles is expected to decrease with decreasing particle size^48^. The low melting point of the Sn or Sn/Si nanoparticles plays an important role in automatic VLS growth without substrate heating; the precipitation and droplet-dot formation of Sn are followed by Si wire growth via continuous irradiation of Si and Sn atoms.

**Figure 5:**
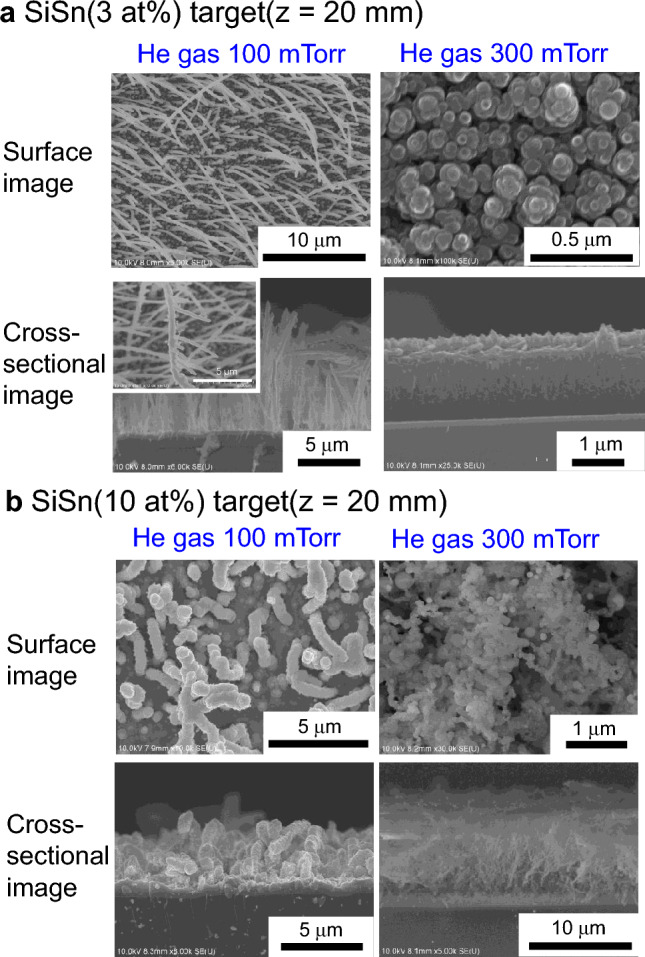
Effect of Sn content in Si/Sn nanowire anodes. SEM surface and cross-sectional images of films deposited with a sputtering-target-to-substrate distance of 20 mm and a He gas pressure of 100 and 300 mTorr. (**a**) SiSn sputtering target with a Sn content of 3 at%. (**b**) SiSn sputtering target with a Sn content of 10 at%.

In addition to the heating effect attributed to He gas, the Sn content also affects the single-step fabrication of the 1D nanostructures. Figure 5a, b show SEM images of films deposited using targets with Sn contents of 3 and 10 at%, respectively. In the films deposited using the 3 at% Sn target and 100 mTorr of He gas, nanowires are distributed with a very low density and clearly grow perpendicular to the substrate surface. This result is reasonable because a smaller amount of Sn atoms in the gas phase leads to a lower density of Sn catalyst dots on the substrate surface, and these dots are the trigger points of nanowire growth. Interestingly, we also found that nanowires clearly branch off and grow perpendicular to a main wire (see the magnified part of the 100 mTorr SEM image in Fig. 5a), which is evidence of secondary VLS growth on the Si-core surface. In our sputtering system, Si and Sn atoms are simultaneously supplied during the deposition process. As a result, the precipitation and droplet-dot formation of Sn, followed by Si-wire growth, occurs repeatedly not only on the substrate surface but also on the grown-wire surface. Thus, the proposed plasma process enables the formation of a fibrous 1D nanowire film with a spider-web-like network by random repetitive VLS growth. As the He-gas pressure was increased to 300 mTorr at a low Sn content of 3 at%, the wire structure was no longer observed; thus, an appropriate combination of He-gas pressure and Sn content is important to transition the nanostructure morphology from 3 to 1D.

By contrast, the nanowire shape markedly changed at the higher Sn content of 10 at% (Fig. 5b); nanowires with a larger diameter and shorter length were formed when the gas pressure was 100 mTorr, whereas aggregation of high-density nanowires and nanoparticles was observed when the gas pressure was 300 mTorr. Abundant Sn atoms in the gas phase led to the formation of Sn dots with a larger diameter or a higher density on the surface, which created two unique wire structures of the extremes described above.

In a similar study involving Sn catalyst dots, Yu et al. reported the growth of Sn-catalyzed VLS Si nanowires via SiH_4_-plasma CVD, where the SnO_2_-film substrate was first treated with H_2_ plasma at 300 °C to form Sn nanodots and then Si atoms from SiH_4_ plasma were supplied to the Sn droplet nanodots on the substrate at 300–600 °C^49–53^. A major difference between our work and that of Yu et al. is the structure of the wires. In their two-step procedure, typical VLS growth resulted in Si nanowires with a vertically aligned structure with a Sn dot at the top. By contrast, in our single-step PVD procedure, random repetitive VLS growth results in Si/Sn composite nanowires with a spider-web-like network structure.

### Performance of Li-ion batteries with Si/Sn composite nanowire anodes

**Figure 6:**
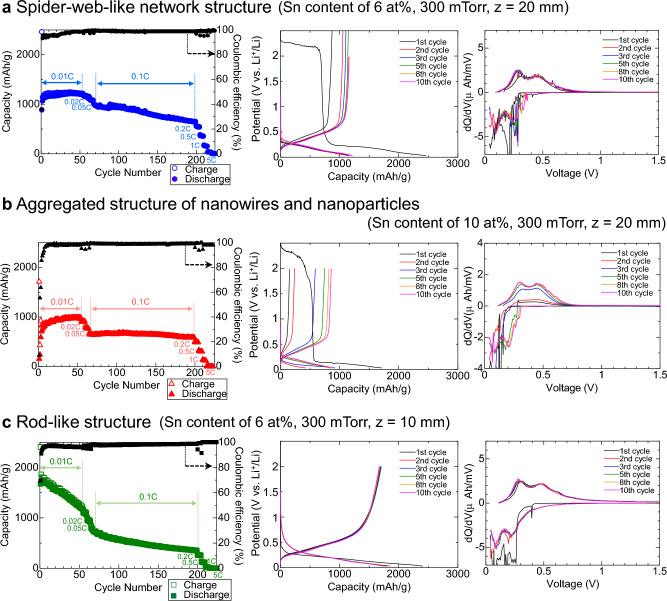
Performance of Li-ion batteries with Si/Sn composite nanowire anodes. Cycle performance, charge–discharge curves, and corresponding d*Q*/d*V* curves for Li-ion batteries. (**a**) Anode of Si nanowires with a spider-web-like network structure. (**b**) Anode of Si nanowires with an aggregated structure of nanowires and nanoparticles. **c** Anode of Si nanowires with a rod-like structure.

We tested the performance of Li-ion batteries with Si/Sn composite nanowire anodes. Figure 6a–c show the gravimetric capacity of a Si/Sn-nanowire film with the spider-web-like network structure, the nanowire–nanoparticle aggregation structure, and the rod-like structure, respectively, as a function of the number of charge–discharge cycles. The gravimetric capacity was calculated by dividing the observed capacity (mAh) of the Li-ion battery by the mass (g) of the Si nanowire film as the active anode material. The charge–discharge current was varied from a 0.01-C to a 5-C rate at certain cycle intervals. Here, a 1-C rate refers to the test current required to fully charge the battery in 1 h; the test current at a 0.01-C rate for a high-capacity Si anode (4200 mAh/g) is approximately the same as that at a 0.1-C rate for a commercial graphite anode (372 mAh/g). First, under a 0.01-C rate for a Si anode (4200 mAh/g), a high gravimetric capacity greater than 1000 mAh/g was observed for all the Si-anode cells, and a Coulombic efficiency greater than 95% was achieved in the first 4–10 cycles. Here, the Coulombic efficiency was calculated as the ratio between the integrated discharge current and the integrated charging current at each cycle. The charge–discharge voltage profile and corresponding differential capacity (d*Q*/d*V*) curves were analyzed in detail for the first 10 cycles. As shown in the voltage profiles (middle graphs of Fig. 6a, b), a plateau region was observed at ~ 2.3 V in the first charge cycle. In general, an organic electrolyte reacts with the surface of a Si anode to form a solid electrolyte interphase (SEI) layer during the initial operation at high voltage^54^. In the Si/Sn composite anodes composed of the narrower 155- and 133-nm diameter nanowires (Fig. 6a, b, respectively), extensive decomposition of the electrolyte proceeds on an abundant wire-surface area for the initial SEI formation, which leads to a continuous high potential in the constant-current first charging process^54,55^. By comparison, no plateau region was observed in the profile for the Si/Sn composite anode with the larger 605-nm diameter nanowires (Fig. 6c); the total surface area of the Si nanowire anode and the related SEI formation process influence the first stable voltage profile without the plateau. To suppress the observed high-potential plateau phenomena, a systematic investigation of the optimal wire diameter and density and the optimal film thickness for stable SEI formation is needed.

In the d*Q*/d*V* curves, distinct peaks appeared at 0.20–0.26 V during Li alloying, and at 0.27–0.30 V and 0.43–0.46 V during Li de-alloying in the first 10 cycles; the phase transformation between amorphous Si and Li_*x*_Si was successfully repeated for all the Si nanowire anodes after the 10 cycles^55–58^. After 10 cycles, the battery cells with the spider-web-like network structure (a) and the wire-particle aggregation structure (b) showed very stable behavior, without capacity fading, for 54 cycles; the capacity retention was greater than 98%. For the cells with a rod-like structure (c), the highest capacity of 1700 mAh/g at cycle 5 decreased to 1174 mAh/g at cycle 54; the capacity retention was 68%. At 65 cycles, the charge–discharge current was increased tenfold (to 0.1-C) and the capacity decreased from 1193 to 1085 mAh/g for the spider-web-like network structure (a), from 973 to 646 mAh/g for the wire-particle aggregation structure (b), and from 1174 to 796 mAh/g for the rod-like structure (c). Finally, the battery cells with anodes prepared using the Si/Sn composite nanowires with the spider-web-like network structure (a), the wire–particle aggregation structure (b), and the rod-like structure (c) showed capacities of 644, 580, and 347 mAh/g after 199 cycles, respectively, and exhibited capacity retentions of 59%, 90%, and 43%, respectively, for 135 cycles at a 0.1-C rate. Notably, the capacity of 580–644 mAh/g observed for the cells with the spider-web-like network and aggregation structures was 1.5–1.7 times greater than that for a commercial graphite anode (372 mAh/g).

To summarize the performance of the Li-ion batteries, the Si/Sn narrower nanowire anode with a spider-web-like network structure (average wire diameter: 155 nm) and the wire–particle aggregation structure (average wire diameter: 133 nm) (Fig. 6a, b, respectively) show stable behavior at a 0.01-C rate for the first 54 cycles. By comparison, the performance of the Si/Sn nanowire anode with a rod-like structure (average wire diameter: 605 nm) markedly decreased with increasing number of charge–discharge cycles. This result is reasonable because McDowell et al. have pointed out that fracture can occur during lithiation of crystalline Si spheres larger than ~ 150 nm in diameter and during lithiation of crystalline Si pillars larger than ~ 300 nm in diameter^59^. When cycled at 0.1-C for 135 cycles, the wire–particle aggregation structure (Fig. 6b) showed the most stable behavior among the three anode materials, with almost no capacity fade. In conclusion, the aggregation structure composed of the narrowest nanowires and smallest nanoparticles used in our experiments showed the most stable cycle performance, with a high capacity of 580 mAh/g at a rate of 0.1-C. However, when the C-rate was increased to 0.2, 0.5, 1, 2, and 5 (left graph in Fig. 6a–c), the capacity decreased to 554, 336, 166, 36, and 1.54 mAh/g for the batteries with the spider-web-like-network structure (Fig. 6a), to 488, 310, 132, 6.95, and 0.7 mAh/g for the batteries with the wire–particle aggregation structure (Fig. 6b), and to 260, 124, 28, 3, and 0 mAh/g for the batteries with the rod-like structure (Fig. 6c), respectively. Further improvements of the nanowire structure and film morphology are necessary to achieve rapid charging in next-generation high-capacity Li-ion batteries.

**Figure 7:**
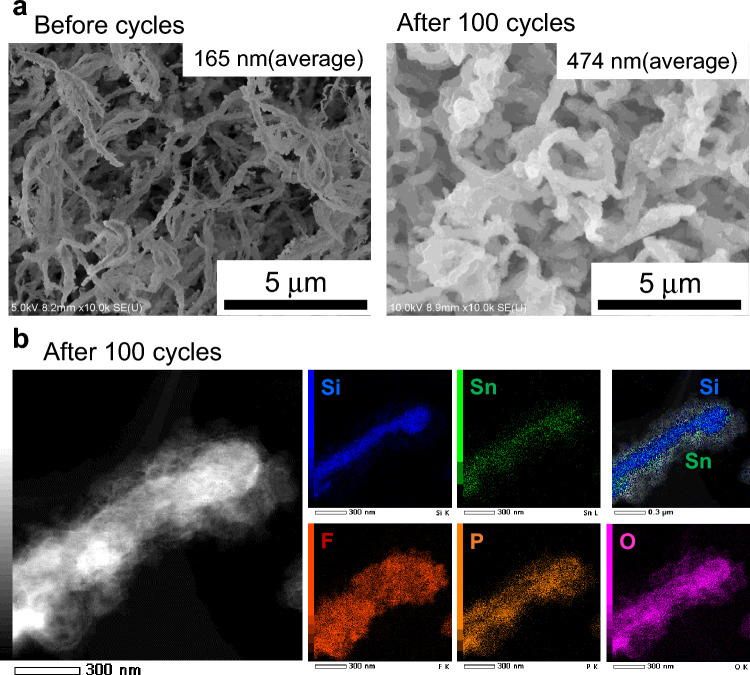
Si/Sn composite nanowire anodes after charge–discharge cycles. (**a**) SEM surface images of the Si/Sn composite nanowire anode with a spider-web-like network structure before and after 100 cycles. (**b**) TEM image and TEM–EDX mapping images of the Si/Sn nanowire anode with a spider-web-like network structure after 100 cycles.

To investigate the mechanism of relatively stable capacity behavior in the Si-nanowire anodes, we analyzed the anode materials after cycling tests by disassembling the cells. Figure 7a show SEM images of a Si/Sn composite nanowire anode with the spider-web-like network structure before and after 100 charge–discharge cycles. The average diameter of the nanowires increased from 165 to 474 nm after 100 cycles as a result of Li alloying. However, substantial pulverization of the Si nanowires was not observed and the physical strain was successfully relaxed by the 2.8-fold radical expansion of the 1D Si nanowires. Figure 7b shows a TEM image and EDX elemental mapping images of wires after 100 charge–discharge cycles, where blue and green indicate Si and Sn atoms, respectively. A Sn layer sill covered the Si wire core after 100 cycles. Notably, another layer was observed on the Si/Sn wire surface; the layer was composed of F atoms (red), P atoms (orange), and O atoms (pink). This layer is reasonably attributed to a chemical reaction between the Si/Sn nanowire surface and the organic electrolyte solution, which is composed of LiPF_6_ dissolved in a mixture of ethylene carbonate (C_3_H_4_O_3_) and diethyl carbonate (C_5_H_10_O_3_). The outer layer is considered part of the SEI layer, although the anode sample was observed by SEM and TEM under the dry condition.

We conducted SEM and TEM analyses of Si/Sn nanowires with a 165-nm diameter in the spider-web-like network structure after 100 cycles. We considered that the wire–particle aggregation structure (wire diameter: 133 nm) was not significantly pulverized because the two anode materials showed similar cycle behavior (Fig. 6a, b). However, the rod-like structure (wire-diameter: 605 nm) might have been pulverized, as evidenced by its substantial decrease in capacity in the first 54 cycles (Fig. 6c), which is similar to the cycle behavior observed for an anode composed of two-dimensional Si thin films, which exhibited material fracture^60–62^.

**Figure 8:**
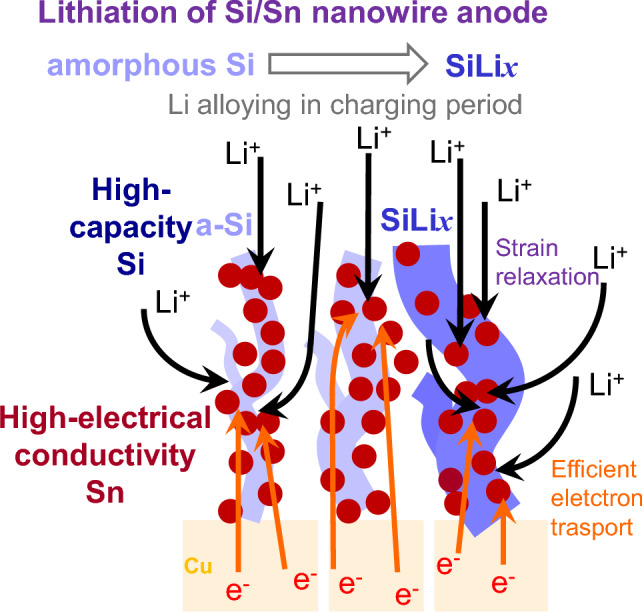
Advantages of the Si/Sn composite nanowire anodes. Schematic of the lithiated Si/Sn composite nanowire anode with a spider-web-like network structure.

The fabricated amorphous Si-core/Sn-shell nanowires have a morphology suitable for Li-ion-battery anodes from mechanical and electrochemical perspectives. To summarize the advantages of the Si-core/Sn-shell nanowires, Fig. 8 shows a schematic of the lithiation of a Si/Sn nanowire anode. First, the amorphous structure of the Si core exhibits more favorable behavior than a crystalline structure when reacting with Li^59^ because amorphous Si nanomaterials without orientation dependence are lithiated isotropically, which reduces the physical stress and suppresses material fracture^59,63,64^. Second, the Sn layer around the Si-core surface enhances the lithiation rate^39,65,66^ because Sn has a high electron conductivity of 10^4^ S/cm and electrons critical for electrochemical reactions (Li alloying) are therefore supplied sufficiently to the Si core from the bottom Cu current collector via the Sn surface layer. Jiang et al. have reported that the electrical conductivity of amorphous SiSn films increases by six orders of magnitude when the Sn concentration in the films is increased from 0 to 10%^43^. We did not measure the electrical conductivity of the Si/Sn composite nanowire anodes. However, the Sn content in the Si/Sn composite nanowire anode was approximately 5–10 at%, which is expected to be sufficient to improve the electrical conductivity of the Si nanowire anode.

**Figure 9:**
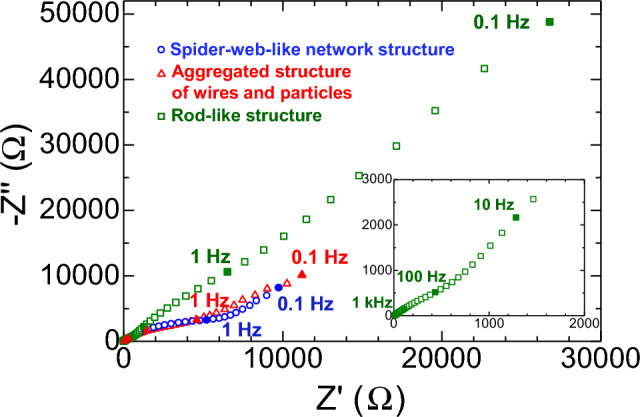
Electrochemical characteristic of the Si/Sn composite nanowire anodes. Nyquist plots of Si/Sn composite anodes, as obtained by electrochemical impedance spectroscopy (EIS), before the charge–discharge process.

Figure 9 shows Nyquist plots of Si/Sn composite anodes, as obtained by electrochemical impedance spectroscopy (EIS), before the charge–discharge cycle. These plots consist of a semicircle in the high-frequency region and a straight line in the low-frequency region. The semicircle in the high-frequency region corresponds to the charge-transfer resistance associated with interfacial Li^+^ ion transfer between the anode and the electrolyte. Among the three investigated anode materials, the rod-like structure (wire-diameter: 605 nm) exhibited the lowest charge-transfer resistance before the charge–discharge cycle. This low resistance contributes to the different electrochemical behavior, which is observed as a lack of a plateau potential in the first charge–discharge voltage profile (Fig. 6c, middle graph). The straight line in the low-frequency region corresponds to the Warburg impedance, which is the impedance associated with ion diffusion. Similar slopes were obtained for the three anode materials, suggesting that ion diffusion within the Si/Sn composite nanowires did not substantially differ among the investigated anode materials^62,67^. In addition, we emphasize that the fibrous spider-web-like network structure of the Si/Sn nanowires is advantageous for a Li-ion-battery anode because, even if one part of the wire is broken, the conductivity of the electrode is preserved by other joints, resulting in high capacity retention.

In conclusion, we fabricated high-capacity Si anodes with various nanostructures in a single-step procedure using plasma sputtering without substrate heating. The film morphology could be controlled from a 3D nanoporous to a 1D nanowire morphology by changing the discharge gas from Ar to He. In particular, 1D nanowire films were obtained under the special sputtering conditions of a SiSn (3–6 at%) target and He discharge gas at a high pressure of 100–500 mTorr. The 1D nanowires consisted of an amorphous Si core with high Li-ion capacity and a crystalline Sn shell with high electrical conductivity, which were suitable structures for Li-ion-battery anodes. The morphology of the Si/Sn nanowire films changed depending on plasma sputtering conditions such as the sputtering target–substrate distance *z* and the Sn content in the Si sputtering target, ranging from (1) a spider-web-like network structure with a wire diameter of 136–257 nm (*z* = 20 mm and Sn content = 6 at%) to (2) a rod-like structure with a rod diameter of 605 nm (*z* = 10 mm and Sn content = 6 at%) and (3) an aggregated structure of nanowires and nanoparticles (*z* = 20 mm and Sn content = 10 at%). We evaluated the charge–discharge cycle performance of the Si/Sn nanowire anodes in Li-ion battery cells. The Si/Sn nanowire anodes with the spider-web-like network structure and the nanowire–nanoparticle aggregation structure showed a high Li-storage capacity of 1219 and 977 mAh/g, respectively, for the initial 54 cycles at 0.01-C and finally 644 and 580 mAh/g, respectively, after 135 cycles at 0.1-C. The developed low-temperature plasma sputtering process enabled the formation of a binder-free high-capacity Si/Sn-nanowire anode in a single step.

## Methods

Some of the following explanations of methods overlap with descriptions in our previous papers^68,69^. The Si anode films were fabricated on a Cu disk using 13.56 MHz radiofrequency (rf) magnetron sputtering. The experimental setup was the same as described in our previous work (see Fig. 4 in Refs.^69^). The Cu disk had a diameter and thickness of 15 mm and 80 μm, respectively, and was placed at the center of the substrate holder. The sputtering target was a polycrystalline intrinsic Si disk (1 inch diameter) with a purity of 99.99% or a Si disk with a Sn content of 3, 6, or 10 at%. An rf power of 15.7 W/cm^2^ (80 W) was supplied to the sputtering target for plasma production. Ar or He gas was supplied from the direction of the target to the substrate holder at a flow rate of 16–94 sccm. The gas pressure was set to a high value of 100 to 500 mTorr. The distance between the target and the substrate holder was 10 or 20 mm. The substrate holder was not heated or cooled during film deposition.

Si films deposited onto the Cu disk under various conditions were assembled into Li-ion battery cells (HS flat cell, Hohsen) as anodes with Li metal counter electrodes with diameters of 16 mm and thicknesses of 250 μm. A polypropylene separator with a diameter of 24 mm and a thickness of 24 μm was placed between the Si anode and Li cathode. A solution of 1 mol/L LiPF_6_ dissolved in a mixture of ethylene carbonate (EC) and diethyl carbonate (DEC) (EC:DEC = 1:1 volume%) was used as an electrolyte in the battery test cells. The battery cycle performance was analyzed at a constant current of less than 1 mA, corresponding to a C-rate of 0.01 to 5, for all the charge–discharge cycles; the cut-off voltage was 0.03–2.0 V. The cycle tests were conducted at room temperature using a battery test system (HJ1001SD8, Hokuto Denko).

To analyze the material properties of the Si anodes, Si films were deposited onto *n*-type Si wafers with a low resistivity under the same sputtering conditions used for the battery Si anodes. The crystal structure was evaluated by XRD (Rigaku SmartLab), and the surface morphology and cross-sectional microstructure were analyzed by SEM (SU-8010, Hitachi) and TEM (JEM-ARM200F, JEOL).

## Data Availability

The datasets used and/or analyzed in this study are available from the corresponding author upon reasonable request.
